# A Scoping Review of the Prevalence of Fascioliasis in Malaysia and Risk Factors for Infection

**DOI:** 10.21315/mjms2020.27.1.3

**Published:** 2020-02-27

**Authors:** Mohamad Ahmad Najib, Noor Jamil Noor Izani, Wan Abdul Wahab Wan Nor Amilah, Arizam Muhamad Faez, Zainuddin Shafizol

**Affiliations:** 1 School of Health Sciences, Universiti Sains Malaysia, Kelantan, Malaysia; 2 Faculty of Veterinary Medicine, Universiti Malaysia Kelantan, Kelantan, Malaysia

**Keywords:** Fasciola spp, fascioliasis, prevalence, risk factors, Malaysia

## Abstract

This review aimed to provide a comprehensive overview of ruminant and human fascioliasis in Malaysia and to identify research gaps in knowledge of the prevalence of fascioliasis in Malaysia and risk factors for the disease using available evidence-based data. We conducted a scoping review based on the framework of Arksey and O’Malley. The preferred reporting items for systematic reviews and meta-analyses were used to guide the review process. The citation search was performed between May and September 2018. Using specific keywords, literature published between 1998 to September 2018 was retrieved from electronic databases. Six articles related to fascioliasis in Malaysia were included in the final review from 1,932 screened articles and reports. Five studies focused on ruminants, including cattle, buffaloes, sheep and goats in the states of Terengganu and Perak. The most frequent ruminant fascioliasis outbreaks involved cattle and goats, with a prevalence of 82%–95% and these outbreaks occurred in Terengganu. Only one study examined the risk of fascioliasis in cattle. In the study, the age and sex of the cattle were important risk-related parameters. The search returned only one documented report of a suspected case of human fascioliasis with an atypical clinical presentation. At present, human fascioliasis in Malaysia remains under-reported and its prevalence remains unknown. The data summarised in this review based on existing evidence identifies research gaps on fascioliasis in ruminants and humans.

## Introduction

Fascioliasis is an important zoonotic parasitic disease that affects animals and humans worldwide (1). The disease is caused by ingestion of encysted metacercariae of liver flukes species *Fasciola hepatica* and *Fasciola gigantica.* These trematodes are leaf-shaped flatworms, large enough to be visible to the naked eye. Both species cause similar disease. The life-cycle of liver flukes is complex. It involves a carrier (i.e. suitable aquatic plants), an intermediate host (where the larval stages of the liver fluke develop) and a final host (where the liver fluke reaches sexual maturity). *Fasciola* infection is transmitted to both animals and humans in the same way (i.e. via food/water contamination). In general, animals are responsible for perpetuation of the infection in the environment.

*Fasciola hepatica* is found in sheep and cattle and is widely distributed in Europe, Africa, Asia, Oceania and North and South America (2). *Fasciola gigantica* is common in cattle and buffalo in tropical zones, including Malaysia (2). Fascioliasis has been reported in ruminants, such as sheep, goats, cattle, buffaloes and camels, as well as herbivores, where the infection rate reaches up to 90% in some endemic areas, such as Bolivia, China, Ecuador, Egypt, France, Iran, Peru and Portugal (3). Worldwide, ruminant and human fascioliasis has markedly increased in endemic areas (4). Several countries in South East Asia, especially Vietnam and Thailand, are considered endemic areas for fascioliasis. The prevalence of bovine fascioliasis in Vietnam was reported to be 23.4% (5), whereas it was 11.8% in both cattle and buffalo in Thailand (6).

To date, the availability of information on the prevalence of fascioliasis in cattle in Malaysia and the risk factors for the disease is very limited and fragmented. Data on the status of ruminant fascioliasis in Iran, Egypt, Thailand and Vietnam can aid disease prevention, control and treatment (5–8). As demonstrated in the literature, fascioliasis is an important veterinary disease, as it causes considerable economic losses in the livestock industry due to the costs of anti-helmintics, drenches, labour and liver condemnation in meat inspections (9). In addition, fascioliasis infection leads to reduced growth rates, decreased fertility, decreased meat and milk production and increased mortality (10).

Ruminant fascioliasis occurs through ingestion of forage containing parasitic metacercariae cysts. Once ingested, excysted metacercariae survive in the small intestine as juvenile flukes. They then move up the gastrointestinal tract, penetrate the host’s intestinal wall and invade the abdominal cavity. Subsequently, they migrate to hepatic parenchyma or bile ducts, causing significant damage (i.e. haemorrhages) by tunnelling through the liver (11). Previous studies identified several risk factors associated with fascioliasis in ruminants (7, 12). These included age, sex, breed, type of farm, temperature and humidity.

As noted above, these parasitic trematodes may infect humans but not directly from ruminants. Infections pre-dominantly occur in rural areas associated with particular types of ruminants rearing (13). In humans, fascioliasis infection occurs through ingestion of fresh water vegetation, namely watercress, on which the parasitic metacercariae encysts and by consumption of contaminated water or the ingestion of food items washed with such water (14). Previous research estimated that about 17 million people worldwide were infected with fascioliasis, with about 91.1 million at risk for infection (1). A high incidence rate of human fascioliasis was reported in the Middle East (Egypt, Iran, Iraq, Syria and Saudi Arabia) and North Africa (Ethiopia) (15) and South America (Peru and the Bolivian Altiplano) (16). In Vietnam, human fascioliasis is common, with a reported prevalence of 7.75% in 2015 (17) and 5.9% in 2016 (18). Since 1990, at least 25 cases of human fascioliasis have been reported in Thailand (19). In Malaysia, only one suspected case of human fascioliasis with an atypical clinical presentation has been reported (20). Human fascioliasis may have serious hepatic pathological consequences in the liver, with severe damage occurring due to migration of the flukes to other organs (21). Human fascioliasis is often misdiagnosed as other clinical complications, such as fasciolopsiasis, which is due to infection by *Fasciolopsis buski* (22*)*.

Ruminant farming activities are increasingly popular among farmers in Malaysia, with hundreds of cattle, sheep, goat and buffalo farms throughout the country. The public health sector in Malaysia views ruminants and human fascioliasis as less important than other parasitic diseases due to the scarcity of epidemiological data on fascioliasis and case reports on disease morbidity and mortality. At present, evidence regarding the risk factors associated with fascioliasis in ruminants and humans varies in different geographical areas (23). More information on risk factors for the disease is needed in geographic regions where research on fascioliasis is limited. An understanding of the epidemiology, disease pathology and risk factors, as well as detection methods, is important in preventing misdiagnosis of *Fasciola* infection among ruminants and humans.

The present review explores evidence on ruminant and human fascioliasis, as well as the local prevalence and risk factors for the disease. It aims to provide a comprehensive overview of ruminant and human fascioliasis and to identify research gaps in the detection and prevalence of fascioliasis in Malaysia, as well as risk factors for the disease, based on available evidence-based data. It is intended to serve as an informative resource for researchers and practitioners to understand the importance of the disease, to recognise associated disease-related factors and to ultimately improve the detection, diagnosis, treatment and control of fascioliasis in Malaysia.

## Materials and Methods

In the present scoping review, the prevalence of ruminant or human fascioliasis was defined as the proportion of ruminants or humans found to be infected with *Fasciola* spp. using various tools for its detection and diagnosis. The risk factor was considered a factor, such as a habit, underlying illness or an environmental condition, that predisposed an animal or individual to develop a particular disease. This scoping review utilised the established scoping review framework of Arksey and O’Malley (24). The scoping review framework included six stages: i) identifying the research question; ii) identifying relevant studies; iii) study selection; iv) charting the data; v) collating, summarising and reporting the results; and vi) consultation with stakeholders and experts in fascioliasis (24).

### Identifying the Research Question

The present scoping review sought to answer the following research questions:

- What is the prevalence of ruminant fascioliasis in Malaysia?
- What is the prevalence of human fascioliasis in Malaysia?
- What are the risk factors for fascioliasis in ruminants?
- What are the risk factors for fascioliasis in humans?

### Identifying Relevant Studies

In this scoping review, we included studies on ruminant and human fascioliasis in Malaysia. A literature search was conducted between May and September 2018 according to the guidelines of the modified preferred reporting items for systematic reviews and meta-analyses (25). Subject headings, lists of keywords, synonyms and MeSH terms (fascioliasis or *Fasciola* or *Fasciola* sp. and Malaysia) were used as search terms by the research team members to identify potential studies (Table 1). An experienced researcher conducted the search, aided by a research librarian. Boolean operators (OR, AND, NOT), including adjacencies and truncations, were used to combine the keywords and related terms during the literature search.

Using established resources, a comprehensive search was performed to identify primary studies and grey literature, including technical reports, on fascioliasis in Malaysia from 1998 to September 2018. These included different electronic databases (MEDLINE, Scopus, EMBASE, EBSCOhost, ScienceDirect, ProQuest and Google Scholar). The World Public Health website and World Health Organization reports were included in the search to retrieve relevant information. A manual search of local Malaysian publications was also performed. This search included publications of the Ministry of Health Malaysia, Ministry of Agriculture and Agro-based Industry Malaysia, *Malaysian Journal of Veterinary Research* and Department of Veterinary Services research article library publications.

### Study Selection

The inclusion criteria for the search were published articles from 1998 to September 2018 to ensure the collection of relevant recent data related to ruminant and human fascioliasis. Narrative, systematic or other review papers were excluded. The study selection was limited to Malay and English language articles.

The selection of articles was performed in two stages. In the first stage, researchers (working in pairs) independently screened the titles and abstracts of all the identified resources based on the inclusion criteria and search terms. Care was taken to ensure that important data were not missed during the selection process. The researchers thoroughly screened the selected titles and abstracts to determine the suitability of the content for inclusion in the review (i.e. met the review’s objectives). Unrelated abstracts were excluded. The researchers then retrieved the full articles of the selected abstracts. In the second stage, the full articles were screened to identify items related to the objectives of the review and to answer the review questions. Similar to the first stage, each pair (two or three researchers) independently reviewed the full articles to determine whether they met the objectives of the review. To ensure study selection consistency, the data collected by the researchers were compared and any discrepancies between the reviewers were discussed. Data management was done using Mendeley software, version 1.19.2 and extracted data from the full articles were documented in a Microsoft Excel spreadsheet.

### Charting of Data

Five reviewers undertook the final full text review on the prevalence of and risk factors for ruminant and human fascioliasis in Malaysia. The researchers developed a standard charting table to categorise the research topics according to two domains: i) the prevalence of and risk factors for ruminant fascioliasis; and ii) the prevalence of and risk factors for human fascioliasis. General and specific information on the studies were included in the charting table. These included the author(s), year of publication, objectives or aims of the study, study location and settings, study population, study design, sample size, sample types, detection methods used, prevalence data and analysis of potential risk factors.

### Collating, Summarising and Reporting the Results

The results of the extracted data were summarised and tabulated in Table 2. We did not assess the quality of the articles, as this was outside the remit of this scoping review. Some limitations of the studies are highlighted to address research gaps and to make useful recommendations for future research on fascioliasis.

### Consultation with Programme Managers and Experts in Fascioliasis

We also conducted consultations with relevant key informants attending the Seminar on Parasitic Infection in East Coast Malaysia 2018 to obtain insights and additional resources and to determine the direction of future research on fascioliasis. The key informants included researchers and experts from local universities and the Malaysian Ministry of Health, Veterinary Research Institute and Department of Veterinary Science Malaysia.

## Results

**Figure 1:**
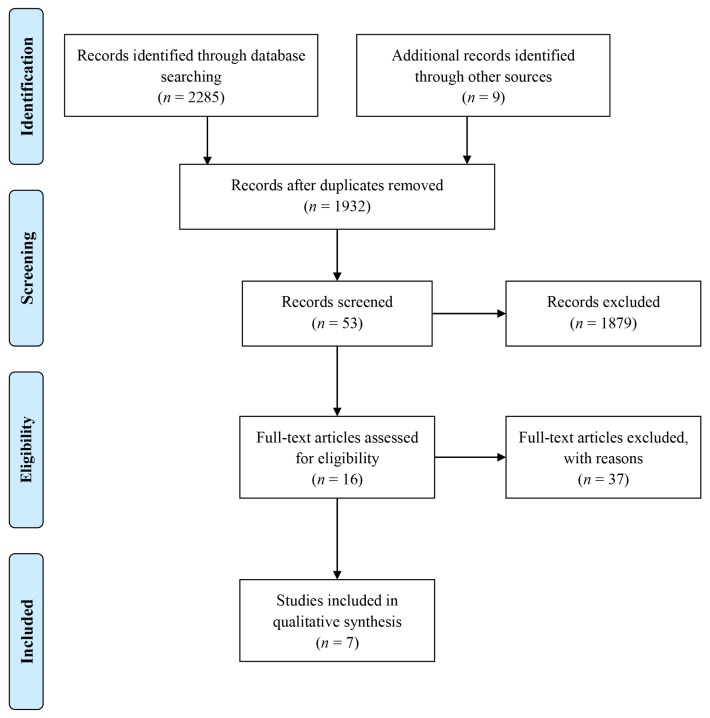
Flow diagram of the scoping review process (25)

In total, 2,294 titles and abstracts were screened during stage 1. Of these, 362 duplicates were removed. The remaining 1,932 articles were screened for abstract eligibility. Only 53 articles were eligible and the remaining 1,879 were excluded. Of these 53 articles, 16 potentially relevant articles were eligible for inclusion in the full text review process. The remaining 37 articles were excluded primarily because they focused on helminth infections other than liver fluke, fascioliasis in countries other than Malaysia, or they were review articles. In the following full-text assessment, 7 of the 16 articles met the criteria for inclusion in this review (Figure 1).

### Study Characteristics

Table 2 provides a summary of the characteristics of the studies. The abstracted data were categorised into study objectives, study design, sample size, sample types, detection methods and study outcomes on the prevalence of fascioliasis and risk factors for the disease. Six studies reported the prevalence of fascioliasis in large and small ruminants, which included cattle, buffaloes, sheep and goats. Four of the six studies were conducted in Terengganu. The other two studies reported the prevalence of ruminant fascioliasis in Perak, Selangor, Kelantan and Pahang.

The designs of the studies varied. They included five cross-sectional studies and one longitudinal study, with sample sizes ranging from 40 to 267 samples. The samples were obtained from 2 local abattoirs and 34 cattle farms. The ruminant samples were stool, blood and liver. The average sampling period ranged between 2 and 10 months during both wet and dry seasons.

There was only one reported indigenous case of suspected human fascioliasis, with ova of *Fasciola* spp. detected in breast tissue of a male patient (20). In this case, the patient had presented to Kuala Lumpur Hospital with a history of liver abscesses and subsequently developed chronic granulomatous mastitis of his right breast, with histopathological findings suggestive of *Fasciola* infection. Although a faecal sample did not show any ova or cysts, a diagnosis of fascioliasis was made based on the morphological characteristics of *Fasciola* ova found in the granulomatous breast tissue sample of the patient.

### Prevalence of Fascioliasis and Risk Factors for Infection

All the included studies on ruminants described the prevalence of fascioliasis. The prevalence of fascioliasis in cattle and buffaloes in Perak was 7.46% and 7.69%, respectively, based on positive detection in condemned liver samples (10). In a longitudinal study, Masrin et al. (26) reported positive *Fasciola* infection in 11.02% of ruminant faecal samples from 2004 to 2013. The prevalence of fascioliasis in faecal samples of cattle in Kuala Terengganu, Terengganu in two separate studies was 95% (27) and 67% (28). Another two studies in Terengganu reported fascioliasis in 82% of cattle (29) and 89% of sheep and goats (30) based on serological analysis. Of these primary studies, only one article described the risk factors for fascioliasis in cattle. According to one study, ruminant age and sex were significantly associated with fascioliasis. In the study, the average age of infected cattle was between 5 and 10 years. The infection rate in female cattle was higher than that in male cattle (29).

The diagnostic tools used in the studies included microscopic examination of liver flukes in condemned liver, copro-microscopic examinations of *Fasciola* eggs and serological tests using an enzyme-linked immunosorbent assay (ELISA). Four of the studies employed the faecal sedimentation technique as a tool to enhance the discovery of *Fasciola* eggs. An ELISA was used in ruminant cases where samples yielded negative results using the faecal sedimentation technique.

Since the one case report of human fascioliasis more than 10 years ago, there have been no new reported cases of human fascioliasis published or reported in Malaysia.

## Discussion

Fascioliasis represents a significant public health threat in several endemic countries, with millions of individuals estimated to be at risk of infection or already infected (1). Fascioliasis disproportionally affects poor individuals, generally in resource-limited regions and farming communities. We summarised the peer-reviewed literature on the prevalence of fascioliasis in Malaysia and risk factors for infection. The findings of the present review have several implications for research practice and policy. First, research activity and information on local prevalence/risk factors in both ruminants and humans seem to be limited. Over two decades, only seven articles, including one human fascioliasis case, reviewed met our inclusion criteria. Due to the lack of information by government agencies on the topic, knowledge of the disease among the public, physicians and farming community is lacking.

Ruminant fascioliasis has been widely reported worldwide and represents a significant economic loss to farmers due to the adverse effects caused by liver fluke infections (7, 31). The reviewed studies in Malaysia did not include extensive epidemiological data on ruminant fascioliasis. Therefore its impact on the economy in terms of the local livestock industry is unclear. Furthermore, the studies included only a few states in Peninsular Malaysia, with no reported surveillance activity in other regions, including states in East Malaysia (Sabah and Sarawak), which have intensive ruminant farming activities. It is clear that comprehensive data on the countrywide prevalence of ruminant fascioliasis is lacking.

Our review highlighted the state of Terengganu as an endemic area of ruminant fascioliasis, with a lower infection rate recorded in Perak. The studies lacked data on factors associated with fascioliasis in ruminants. Only one study focused on the risk factors for infection in ruminants (29). In the study, ruminant age and sex were significantly associated with fascioliasis. Although there was a consensus among the included studies that age and sex were significant risk factors for fascioliasis in ruminants, researchers in Malaysia do not appear to have addressed the association of socio-demographic factors or causative factors with infection. However, socio-demographic factors were associated with the prevalence of infections in ruminant studies in other countries, such as Egypt and Bangladesh (7, 32).

The study in Perak was based on liver condemnation of samples from local abattoirs. The studies in Terengganu were based on liver condemnation, microscopic observations of stool samples that had been collected from farmed cattle using the formalin ether sedimentation technique and antibody detection of serum samples using an ELISA. The review highlights the heterogeneity in sampling and diagnostic tools used for screening of the infection. Such studies are prone to diagnostic bias, as samples from farmed cattle are more likely to be positive for fascioliasis than samples collected in an abattoir (10, 27, 29, 30). The reduced rate of positivity in abattoir is because farms which are involved in scheduled veterinary inspection usually provide the abattoir with well nourish and healthy cattle. In addition, an ELISA is more sensitive and specific (> 95%) than copro-microscopic analysis of stool samples (33). The aforementioned factors cast doubt on the accuracy and validity of the studies that summarised the prevalence of fascioliasis infection based on microscopic observations of stool samples.

Our review also highlighted the lack of data on the prevalence of ruminant fascioliasis using molecular detection methods. Most of the studies summarised in this review used conventional approaches (i.e. microscopic detection of parasite eggs in stool samples and antibody detection using an ELISA). According to a recent study, the specificity of molecular methods, such as the polymerase chain reaction and loop-mediated isothermal amplification, in diagnosing fascioliasis is comparable to that of an ELISA but both had poor sensitivity compared to ELISA (34). Although serological methods enable early detection of fascioliasis, circulating antibodies can remain in blood for several months after successful deworming (35). Thus, serology measures only exposure to the parasite and is not always an indicator of current infection. Distinguishing a significant and current infection from past disease exposure is crucial in reporting accurate epidemiological data.

In the present review, the sample size in several of the studies was fewer than 100. The small sample sizes may have influenced the results and led to discrepancies in terms of the reported prevalence. For example, a small sample size in an endemic area may yield a higher infection rate as compared with that of a large sample size in the same area (36). We also identified issues related to the duration of sampling. None of the studies attempted to determine the seasonal occurrence pattern of ruminant fascioliasis, although such patterns are possible. Some previous studies reported occasional cases of fascioliasis in cattle during both the wet and dry seasons although the disease was more commonly associated with the wet season (37). The seasonal pattern of fascioliasis during both dry and wet seasons needs to be analysed to address the effect of climate change on disease transmission.

Ruminant farming is increasingly popular in Malaysia and farmers are at risk of infection. However, except for one case reported in 2006, there have been no cases of human fascioliasis recorded in Malaysia to date. Some cases of human fascioliasis have been reported in Asia (38, 39). Over the past 25 years, human fascioliasis has also been recorded in Iran, China (40) and Vietnam (5). A plausible reason for the rare occurrence of the disease in humans in Malaysia is the absence of extensive evaluations of the status of fascioliasis in humans. Given that more than half of human cases of fascioliasis are subclinical and a fluke is retrieved from a patient only rarely, human infection may be difficult to diagnose. Based on the findings of the present review, there is limited evidence for human fascioliasis at present. The absence of human exposure to sources of infection, together with the lack of an appropriate diagnostic approach and a low suspicion among medical practitioners of fascioliasis in patients, may contribute to the apparent rarity of the disease in Malaysia.

In areas where the disease occurs sporadically, fascioliasis affects in all age groups. In contrast, in areas where the infection is highly endemic, the infection tends to be more common among school-aged children. People living in rural areas also typically have a higher risk of infection than those living in urban areas. However, as fascioliasis in humans can be transmitted through ingestion of aquatic vegetation encysted with metacercariae and drinking water contaminated with free-living metacercariae (1), human cases of fascioliasis infection may occur anywhere. According to a previous study, infection is linked to the consumption of particular aquatic plants, which are part of the regular diet of particular group of people in many countries (21).

*Fasciola hepatica* and *Fasciola gigantica* live in the biliary tracts and gallbladders of humans and cause histopathological damage to these organs. Previous studies provided evidence of extra-hepatic fascioliasis in different organs in humans, especially in known endemic areas (41–44). Studies also reported confirmed findings of juvenile *Fasciola* spp. migration (45, 46) and gravid *Fasciola* in some organs based on observations of tissue-embedded ova (43, 47–49).

Awareness among researchers and medical practitioners of human fascioliasis needs to be increased to ensure that fascioliasis is included in differential diagnoses, thereby preventing related complications and disease spread. In this regard, clinical manifestations, imaging and laboratory findings are important. Clinical manifestations of acute fascioliasis include abdominal pain, fever and constitutional symptoms, together with evidence of the risk of possible infection. Laboratory evaluations revealing eosinophilia, leukocytosis and positive findings of infection on ultrasound or computed tomography scanning should increase the suspicion index of the disease. In the differential diagnosis of fascioliasis, similar diseases manifestation such as intraperitoneal and tumours should also be considered. To ensure that fascioliasis does not remain a neglected disease, increased research is needed in this area.

Unlike the treatment with other helminth infections, treating fascioliasis with anti-helminthic drugs, such as praziquantel, has limited effectiveness. Albendazole and mebendazole are not effective in treating fascioliasis. At present, triclabendazole is the drug of choice in the treatment of ruminant and human fascioliasis (50–52). Triclabendazole is effective against all stages of fascioliasis, with a cure rate of more than 90% after two oral treatments of 10 mg/kg (53). In the same study, a reduction in egg number of almost 100% was observed after two treatments. Furthermore, triclabendazole is relatively well tolerated and does not induce side effects (54). However, triclabendazole is not widely available locally. To date, there are no alternatives to triclabendazole for the treatment of fascioliasis (55). In addition, cases of triclabendazole resistance in ruminants (56) and humans (triclabendazole-resistant human *Fasciola hepatica*) have been reported (57). Based on our consultation with programme managers and experts in fascioliasis, the majority of local livestock breeders use ivermectin, an anti-helminthic drug, to treat ruminant helminth infections, including fascioliasis. Ivermectin was used for deworming, as well as for preventing scabies in ruminants. In Malaysia, the drug is available from the district veterinary departments.

The strengths of this review lie in the systematic search of bibliographic databases and independent screening by five reviewers. However, we did not include unpublished conference proceedings or theses in the search because the conference proceedings and theses were not deposited in the available databases. Even though we provide an extensive discussion based on data from seven local studies, this review does not provide an extensive and comprehensive review of the true situation of the prevalence of fascioliasis and risk factors for infection throughout Malaysia, The information gleaned from the small number of studies is insufficient and too fragmented to draw conclusions on the prevalence of fascioliasis in Malaysia and risk factors for infection. Thus, we propose that extensive research is needed to estimate the burden of the disease in Malaysia. We recommend active participation from the veterinary, agriculture, and higher education sectors in carrying out research in this area.

## Conclusion

Our review highlights that the epidemiology of ruminant and human fascioliasis in Malaysia remains unclear. Most likely, the prevalence of fascioliasis is underestimated due to the lack of local and extensive surveys performed in potentially endemic areas. Therefore, the status of fascioliasis in Malaysia remains unknown. A surveillance programme in all states in Malaysia is important, especially to identify the risks associated with animal and human infections, as well as to identify effective disease-prevention measures. Although only one case of human fascioliasis has been reported, screening of fascioliasis in the farming community is important to provide information on the current status of the infection and prevent disease-related complications. The findings can aid future studies to improve the availability of diagnostic facilities and treatment through extensive surveys and research performed in potentially endemic areas with the involvement of veterinary, agriculture and universities, and provide beneficial and effective health policy decision making.

## Figures and Tables

**Table 1:** Keywords and terms that were used in the database search

Keyword 1	Keyword 2	Keyword 3	Keyword 4
Prevalence	Risk factors	Fascioliasis	Malaysia
Epidemiology	Associated factors	*Fasciola* spp.	
Incidence rate		*Fasciola*	
Occurrence		Sheep liver fluke	
		Human liver fluke	
		Liver fluke	
		Ruminant fluke	
		Zoonotic diseases	
		Helminthiasis	
		Human trematode	
		Ruminant trematode	

**Table 2:** Summary of study characteristics (*n* = 6)

Authors (reference)	Objective	Study design and sample size	Sample and detection methods	Prevalence and risk factors
Zainalabidin et al. (10)	Screening of zoonotic fascioliasis on a slaughtered large ruminants in abattoirs in Perak	Cross-sectional studies 80 fresh liver samples were collected from local abattoirs in Ipoh 67 cattles from Kedah-Kelantan breed 13 buffaloes from Murrah breed	Liver sample Microscopy examination on liver condemnation Fluke identified using morphometric methods.	Prevalence: 6 samples (7.50%) were diagnosed with fascioliasis 5 samples from cattle (7.46%) 1 sample were positive for buffaloes (7.69%)
Khadijah et al. (27)	Study on endo- and ectoparasitic infections in two cattle farms located in Kuala Terengganu	Cross-sectional studies 40 rectal faecal samples were collected from two cattle farms in Terengganu: Felda Belara (A) and Kampung Beladau Kolam (B) Sampling duration: September–October 2012	Faecal sample Faecal sedimentation technique	Prevalence: From 37 samples (three animals had no faeces during sampling), 35 samples (95%) were positive for liver fluke eggs All cattle in farm A (*n* = 18) were positive for fascioliasis 17 cattle in farm B (*n* = 19) were positive for fascioliasis
Masrin et al. (26)	Study on status of fascioliasis from 2004–2013 in Veterinary Research Institute Ipoh	Retrospective studies Cases received from district in Perak, Selangor, Kelantan and Pahang	Faecal sample Coprological examination Faecal sedimentation technique Fecal egg count (FEC) method	Prevalence: Percentage of fascioliasis from 2004–2013 (positive/suspected * 100) 2004: 2.4% 2005: 1.2% 2006: 7.1% 2007: 15.2% 2008: 1.4% 2009: 0% 2010: 0% 2011: 0% 2012: 42.9% 2013: 40% Average of positive cases for a fascioliasis from 2004–2013 is 11.02%.
Khadijah et al. (28)	*Fasciola* and *Paramphistomum* infection in large ruminants	Cross-sectional studies A study was conducted in 6 cattle farms and 1 abattoir located in Kuala Terengganu, Malaysia Collection of 60 faecal samples from 6 cattle farms Liver condemnation of 231 cattle and 110 buffaloes in abattoir Kuala Terengganu Sampling duration: December 2014–March 2015	Faecal sedimentation technique Microscopy examination on liver condemnation	Faecal sedimentation technique 67% of farmed cattle positive for fascioliasis Microscopy examination on liver condemnation 3% positive for fascioliasis
Mursyidah et al. (30)	Study on *Fasciola* and *Paramphistomum* infections in small ruminants (sheep and goat) in Terengganu.	Cross-sectional studies Sampling was conducted on 16 farms in Terengganu Districts involve were Besut, Setiu, Kuala Terengganu, Hulu Terengganu, Marang, Dungun and Kemaman Collection of 267 rectal faecal samples from sheep and goat: 41 males and 226 females Sampling duration: March 2015–December 2015	Faecal sample Coprological examination Faecal sedimentation technique Blood sample Serological test using ELISA antibody detection	Faecal sample No detection of *Fasciola* egg in any samples (*n* = 267) Serological test Serum samples were randomly selected for serological screening (*n*_total_ = 86) 89% of the samples (*n* = 76) were positive for *Fasciola* antibody infection
Rita et al. (29)	Study on the prevalence of helminthiasis in cattle, Terengganu	Cross-sectional studies Sampling was conducted on 16 farms in Terengganu Districts involves were: Kuala Terengganu, Hulu Terengganu, Besut, Dungun, Kemaman, Marang and Setiu. Collection of 219 fresh faecal samples: 44 males and 175 females and 214 blood samples Sampling duration: March 2015–January 2016	Faecal sample Coprological examination Faecal sedimentation technique Blood sample 85 samples which yield negative result from coprological examination were tested for serological test using ELISA (*n* = 85)	Faecal sample From 219 samples, 120 samples are positive for trematode infection 49 samples (41.0%) were positive for liver fluke Faecal egg count (FEC) for liver fluke ranged between 0–104 eggs per gram 12 out of 16 farms (75%) were positive for trematode infection Serological test From 85 samples, 82.0% is positive for liver fluke infections Age group 87.0% infection on 5–10 years of age group Significant difference egg between fecal count and age of animal χ^2^ (2, *N* = 122) = 144.1, *P* < 0.05 Gender 55.0% infections in female 36.0% infections in male Significant difference of between gender cattle and prevalence of fascioliasis χ^2^ (1, *N* = 122) = 57.1, *P* < 0.05 District All district in Terengganu is positive for bovine fascioliasis except in Hulu Terengganu The prevalence of fascioliasis is significantly differ across the district χ^2^ (6, *N* = 219) = 163.0, *P* < 0.05
Naresh et al. (20)	Liver fluke of the breast in a male patient	A case report	Malaysian of Pakistani origin, born and bred in Malaysia 56-year-old Male Married with six children Work as a bus conductor No history of travel to endemic areas of fascioliasis Lives in urban neighbourhood Has previous history of liver abscess	Diagnosis of granulomas tissue samples revealed scattered well-defined ova which were identified as ova of the *Fasciola* species
